# Early Serologic Diagnosis of *Mycoplasma pneumoniae* Pneumonia: An Observational Study on Changes in Titers of Specific-IgM Antibodies and Cold Agglutinins

**DOI:** 10.1097/MD.0000000000003605

**Published:** 2016-05-13

**Authors:** Sung-Churl Lee, You-Sook Youn, Jung-Woo Rhim, Jin-Han Kang, Kyung-Yil Lee

**Affiliations:** From the Department of Pediatrics, College of Medicine (S-CL, Y-SY, J-WR, J-HK, K-YL), The Catholic University of Korea, Seoul; and Department of Pediatrics (Y-SY, J-WR, K-YL), The Catholic University of Korea Daejeon St. Mary's Hospital, Daejeon, Republic of Korea.

## Abstract

There have been some limitations on early diagnosis of *Mycoplasma pneumoniae* (MP) infection because of no immunoglobulin M (IgM) responses and variable detection rates of polymerase chain reaction in the early stage of the disease. We wanted to discuss regarding early diagnostic method using short-term paired titration of MP-specific IgM and cold agglutinins (CAs) in the early stage of MP pneumonia.

The participants of this study were 418 children with MP pneumonia during 2 recent epidemics (2006–2007 and 2011), and they were diagnosed by an anti-MP IgM antibody test (Serodia Myco II) examined twice during hospitalization at presentation and around discharge (mean of 3.4 ± 1.3 days apart). CA titers were simultaneously examined twice during study period. Anti-MP IgM antibody titer ≥1:40 and CA titer ≥1:4 were considered positive, respectively. The relationships between 2 IgM antibodies in the early stage were evaluated.

Regarding MP-specific antibody titers, 148 patients showed a seroconversion, 245 patients exhibited increased titers, and 25 patients had unchanged higher titers (≥1:640) during hospitalization. The median MP-specific antibody titers at each examination time were 1:80 and 1:640, respectively; those of CAs were 1:8 and 1:32, respectively. Illness duration prior to admission showed a trend of association with both titers, and patients with shorter illness duration had a higher rate of negative titers or lower titers at each examination time. CAs and MP-specific antibody titers were correlated in the total patients at presentation and at 2nd examination (*P* < 0.001, respectively), and the diagnostic corresponding rates of CAs to IgM antibody test were 81% to 96% in patient subgroups.

Short-term paired MP specific-IgM determinations in the acute stage may be used as a definitive diagnostic method for MP pneumonia. Paired CA titers showed a correlation with MP-specific antibody titers, suggesting they can be used as an adjuvant diagnostic method.

## INTRODUCTION

*Mycoplasma pneumoniae* (MP) is one of the major pathogens causing pneumonia in children and young adults and MP pneumonia appears as a cyclic epidemic disease with a 3 to 7-year interval worldwide.^1,2^ The early diagnosis of MP pneumonia may be important for deciding the treatment modality including the choice of proper antibiotics.

After the onset of systemic symptoms of MP infection, such as fever, sore throat, and myalgia, MP-specific immunoglobulin M (IgM) antibodies are produced first, followed by specific IgG antibodies in the early stage of MP pneumonia. However, there is a time-gap of several days or longer between the appearance of pathogen-specific IgM antibodies and the disease onset. The “gold standard” for serologic diagnosis is a ≥4-fold increase of MP-specific IgG titer 2 to 3 weeks after the initial measurement,^1–3^ but it is not helpful for clinical practice and patient collection for MP infection studies. Moreover, certain diagnostic kits, including complement fixation test, can detect cross-reactive IgG of other mycoplasma strains or other substances.^3^ On the other hand, polymerase chain reaction assays are used for the early diagnosis of MP infection, but they also have some limitations because of the existence of long-term MP carriers in epidemics,^4,5^ different detection rates with respect to disease stage, patient age, sampling sites or possibly causative strain subtypes, and a lack of presentation of definitive evidence of a systemic immune reaction.^6–9^ MP-specific IgM antibodies and other IgM antibodies such as cold agglutinins (CAs) may begin to appear within a week after the disease-onset and increase steadily until peaking during the acute and/or early convalescent stage of MP infection. Therefore, it has been suggested that short-term follow-up titration of IgM antibodies could help obtaining a serological confirmation by seroconversion or the elevated IgM titer within a relatively short-period after the disease onset, that is, during hospitalization.^9,10^

CAs are well known to be elevated in MP infection; accordingly, the CAs test was used as a diagnostic tool for MP pneumonia before the development of specific serologic diagnostics.^11,12^ In addition, CAs positivity is correlated with MP-specific antibody positivity in patients with MP pneumonia.^13^ Since the 2003 nationwide MP pneumonia epidemic, we have used an MP-specific serologic test (microparticle agglutination assay, Serodia Myco II, Fujirebio, Tokyo, Japan) together with the CAs test for the diagnosis of MP pneumonia performed twice during admission.^9,14–16^

Accordingly, the present study evaluated the changes of MP-specific IgM antibodies and CAs as nonspecific IgM antibodies. We also evaluated the relationship between their titers in the early stage of MP pneumonia on the basis of twice examinations during admission. Finally, we briefly discuss the clinical implications of CAs in MP infection.

## METERIALS AND METHODES

### Study Participants and Design

**FIGURE 1:**
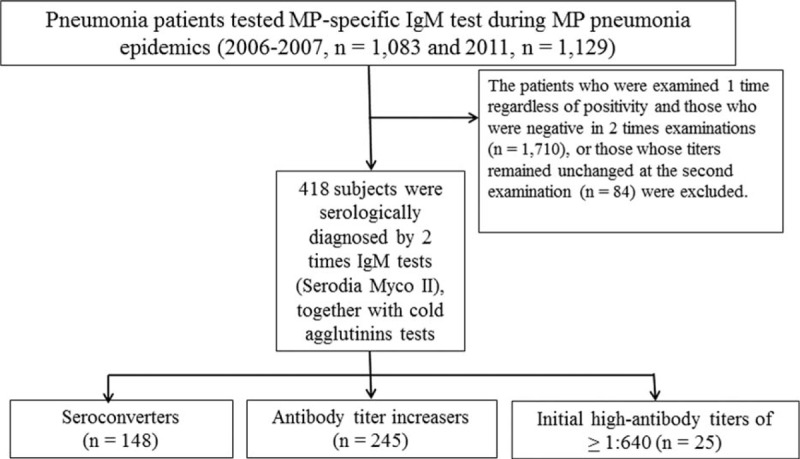
Flow diagram of the patients selected in the study.

In South Korea, MP pneumonia epidemics have been occurred with 3 to 4-year intervals.^16^ The retrospective study was conducted in a general hospital that has 670 beds for children and adults in Deajeon, Korea. The participants of this study were 418 children with MP pneumonia whose diagnosis was confirmed using a serologic MP-specific IgM test (Serodia Myco II) performed twice during hospitalization at presentation and before discharge. The patients were selected during recent nationwide MP pneumonia epidemics occurred in 2006 to 2007 (n = 199) and 2011 (n = 219) in Daejeon, South Korea. All patients exhibited a seroconversion (from negative titer at presentation to positive titer at the 2nd examination, n = 148) or increased antibody titers during admission, n = 245). Among the patients with titers ≥1:40 but ≤1:320 at admission, those whose titers remained unchanged at the 2nd examination were excluded because of the high possibility of past recent infections during the epidemics (n = 84). However, the patients who showed a titer ≥1:640 at admission that remained unchanged at the 2nd examination were included as a subgroup (n = 25) (Figure 1).

### Titration of MP Specific-IgM Antibodies and Cold Agglutinins

Although CAs test was not a routine diagnostic tool for MP infections in Korea as well as in other countries, all patients underwent the CA test twice during hospitalization according to our diagnostic policy for MP pneumonia in place since the 2003 epidemic.^14–16^ For the early diagnosis, the 2nd examination was performed 3 to 4 days after 1st examination before discharge, especially in 2011 epidemics. Some severely affected patients (15 cases) received 3rd or more examinations for the confirmation of seroconversion, and they were included in the subjects. MP-specific IgM and CAs titers ≥1:40 and ≥1:4 were regarded as positive, respectively. Hospitalization stay (in days) was defined according to the admission and discharge dates. The interval between the 1st and 2nd examinations (days) was calculated as the date of the 2nd examination minus the date of the 1st examination. As each patient had a different time of fever and/or cough onset before admission (mean 5.6 ± 2.6 days), we evaluated both antibody titers according to the time of symptom initiation. We divided the patients into 8 subgroups at presentation and the 2nd examination, respectively: 8 subgroups consisted of the patients with different duration of illness from the 2 to 7 days (6 subgroups in each day), the 8 to 13 days, and the ≥14 days. Eight subgroups in the 2nd examination consisted of the patients with illness duration from the 5 to 10 days (6 subgroups in each day), the 11 to 13 days, and the ≥14 days. We evaluated the relationship between MP-specific antibodies and CAs as nonspecific IgM antibodies in total of MP pneumonia patients.

Written informed consent was obtained from the parents/guardians of all children for the use of their medical records. This study was approved by the Institutional Review Board of The Catholic University of Korea, Daejeon St. Mary's Hospital (DC14RISI0030).

### Statistical Methods

Data are presented as mean ± SD or median (range) for skewed data. The relationships between MP-specific antibody and CAs titers of total patients at admission and the 2nd examination were used by Spearman correlation analysis (SPSS ver. 14.0, SPSS Inc., Chicago, IL). A *P* value of <0.05 was considered statistically significant.

## RESULTS

### Clinical Characteristics of Total Patients

The mean age of the patients was 5.4 ± 3.3 years (range: 0–15 years), and the male to female ratio was 1:1 (211:207). Nearly all patients had cough (98.1%), fever (91.6%), and pneumonia lesions in chest radiography (100%) at presentation. The mean hospitalization stay (days) was 6.5 ± 1.9 days and the mean interval between the 2 laboratory examinations was 3.4 ± 1.3 days. The mean durations from initial symptoms such as fever and/or cough to admission and the 2nd examination were 5.6 ± 2.6 days (range: 1 day to 4 weeks) and 9.0 ± 2.8 days (5 days to 4 weeks), respectively.

### Changes of MP-Specific Antibody Titers and CAs Titers During Admission

**FIGURE 2:**
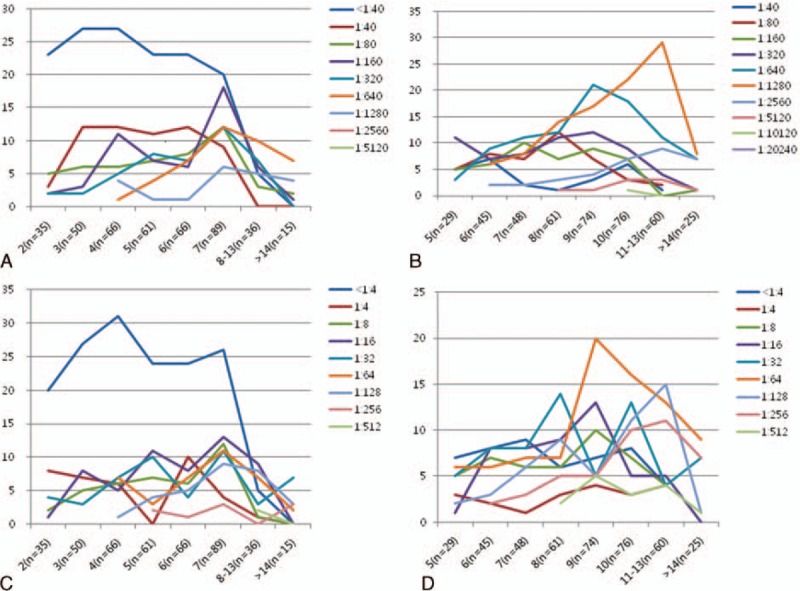
Distribution of MP specific IgM antibody titers at presentation (A) and at 2nd examination (B), and CAs titers at presentation (C) and at 2nd examination (D) according to the duration of illness before admission ^∗^X-axes express the illness duration of patients at the examinations (patient numbers in respective durations of illness), and Y-axes express the numbers of patients who have respective titers of MP specific IgM (A and B) and respective CAs titers (C and D). CA = cold agglutinin, MP = *Mycoplasma pneumoniae*.

The distribution of titers of MP-specific antibodies and CAs is shown in Table 1. Regarding MP-specific antibody titers, 148 patients showed a seroconversion from negative titer (<1:40) at presentation to ≥1:40 at the 2nd examination (range: 1:40–1:5120); 245 patients with positive titers at presentation showed increased titers in the 2nd examination (range: 1:40–1:20,480, respectively) and most of them showed a ≥4-fold increase; 25 patients showed unchanged high titers (≥1:640) in both examinations. The median titers of total patients at presentation and the 2nd examination were 1:80 and 1:640, respectively. Meanwhile, regarding CAs, 157 patients showed a negative titer (1:1), and 261 showed a titer ≥1:4 at presentation. The median titers at presentation and the 2nd examination were 1:8 (range: 1:1–1:512) and 1:32 (range: 1:1–1:512), respectively. The distribution of MP-specific antibody titers with respect to the time of disease onset at presentation is shown in Table 2 and Figure 2A. We analyzed the distribution of MP-specific antibody titers with respect to the time of symptom initiation at each examination. The duration of illness showed a trend of being associated with both titers, that is, patients with a shorter duration of illness had a higher rate of negative titer or lower titers at each examination time (Table 2, Figure 2A and B). The subgroup with 7-day fever duration at presentation was largest (n = 89) and had a relatively even distribution of patients with a broad range of antibody titers from negative to 1:1280, and the subgroups with fever duration of 7, 8, and 9 days at the 2nd examination exhibited similar characteristics. Similar findings were also observed regarding the CAs titers in both examinations (Figure 2C and D).

**TABLE 1:**
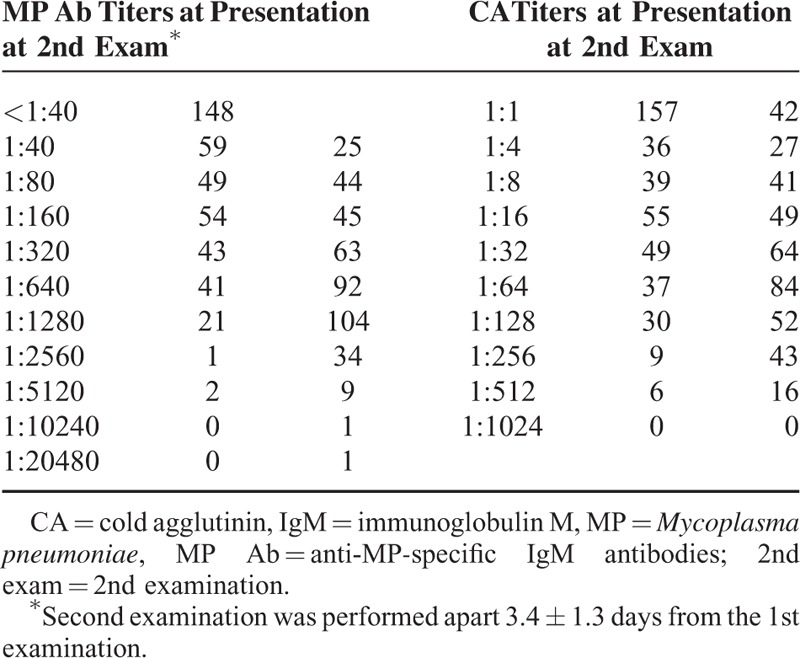
Distribution of Titers of MP Specific-IgM Antibodies and CAs at Presentation and at 2nd Examination (n = 418)

**TABLE 2:**
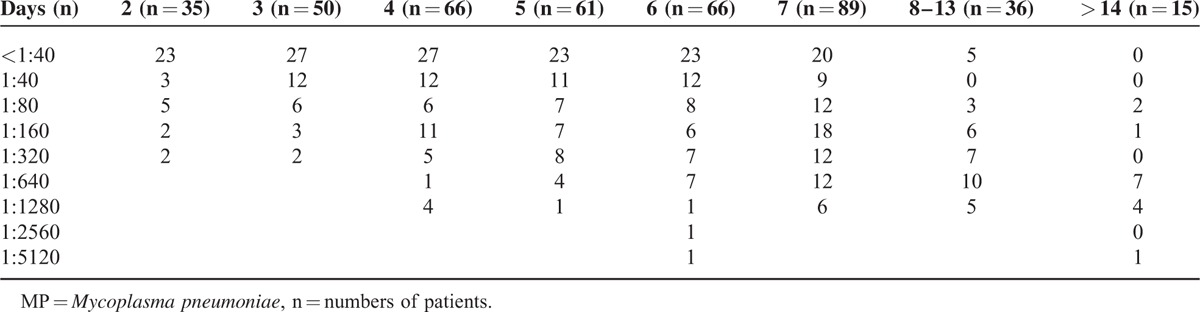
Distribution of Titers of MP*-*Specific Antibodies at Presentation According to the Duration of Illness

### Correlation Between Titers of CAs and MP-Specific Antibodies During Admission

**FIGURE 3:**
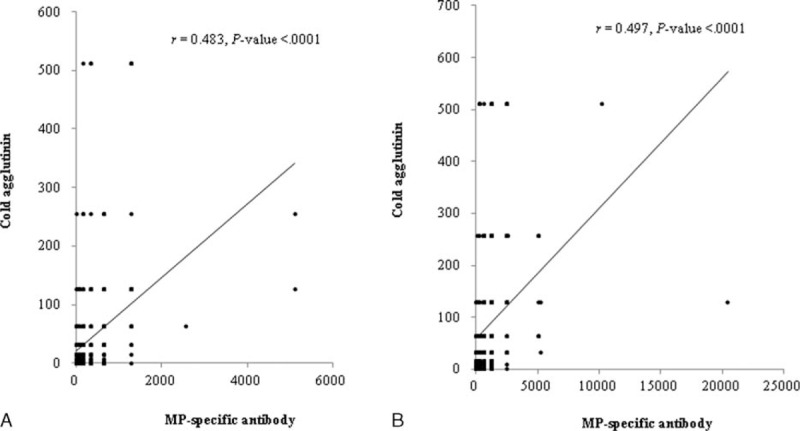
Correlation between titers of MP specific-IgM antibodies and titers of cold agglutinins at presentation (A) and at 2nd examination (B), assessed by Spearman correlation analysis. MP = *Mycoplasma pneumoniae*, IgM = immunoglobulin M.

Although each patient might have different immune reaction against MP infection with different stage of illness, there were significant correlations between MP-specific IgM titers and CAs titers evaluated by Spearman correlation analysis (Figure 3A and B). In addition, we evaluated the corresponding rates of CAs titers for the diagnosis of MP infection, if it were regarded that the MP specific-IgM method in the present study has 100% accuracy. In 2 times MP-specific IgM examinations, 148, 245, and 25 patients exhibited seroconversion, the increased antibody titers, and the unchanged high antibody titers (≥1:640), respectively (Figure 1). Meanwhile, regarding CAs titers, 115 patients achieved seroconversion, 176 had increased antibody titers, 57 remained unchanged, 28 showed decreased titers, and 42 showed negative titers (1:1). Thus, approximately 10% of MP pneumonia patients (42/418) might be considered to have not induced a CA-associated immune response. Among the 57 patients with an unchanged CA titer, 47 and 31 had titers ≥1:32 and ≥1:64, respectively. Among the 148 patients who achieved MP seroconversion, 64 achieved CAs seroconversion, 46 showed increased titer, 11 remained unchanged (*n* = 8 with the same titer of ≥1:32), 7 showed decreased titers (n = 2 ≥1:32 at 1st examination), and 20 were negative (1:1). Among the 245 patients who showed an increase in MP-specific antibody titer, 51 achieved CA seroconversion, 122 showed increased antibody titers, 31 remained unchanged (n = 22, ≥1:32), 20 showed decreased titers (n = 11, ≥1:32 at first examination), and 21 were negative (1:1). Among the 25 patients with the same MP-specific antibody titers (≥1:640), all except one showed a titer ≥1:32 at the 1st examination, 15 remained unchanged, 8 showed an increased titer, 1 patient showed a decreased titer, and 1 case was negative. Together with the pattern change of CAs titers, the titer ≥1:32 at the 1st examination was regarded as positive, the diagnostic values of CAs test to MP-specific IgM diagnosed patients were 81.1% (120/148) in MP-specific IgM seroconverters, 84.1% (206/245) in the titer increasers, and 96% (24/25) in those who had unchanged high titers, respectively.

## DISCUSSION

In MP pneumonia, CAs usually appear by the end of the 1st week of fever onset and disappear by 2 to 3 months.^11,12^ MP-specific IgM antibodies may exhibit a similar pattern but can persist for several months or longer.^3^ In the present study, MP pneumonia patients exhibited a classical immune reaction to substances from insults after MP infection, reflected by the changing titers of MP-specific IgM antibodies and nonspecific IgM CAs during the acute stage of the disease. However, approximately 10% did not exhibit a CA-inducing immune reaction in MP-specific IgM positive patients. In addition, some MP pneumonia patients showed a positive CAs titer before a positive MP-specific antibody titer in the early stage of the disease, with occasional high titers; meanwhile, some patients who had high initial titers (≥1:640 for MP-specific antibody or ≥1:32 for CAs) had the same titers in the short-term follow-up examination. This finding suggests that MP-specific IgM antibody titers and the CA titers can peak during the early stage of the disease in some patients. As immune responses to the insults of MP infection vary among patients, it is natural that the kinds of antibodies, peak antibody titers, and duration of antibody detection may also differ. In MP pneumonia epidemics, many asymptomatic or mild MP-infected patients without pneumonia show positive titers of MP-specific IgM antibodies that can be persist for long time.^1–3^ However, it is unknown whether asymptomatic patients also show CAs-associated immune responses as well as in MP pneumonia patients.

The reason for the appearance of CAs during MP infection remains unknown. CAs are IgM antibodies that can bind to ligands, specifically I antigens on red blood cell (RBC) surfaces, and induce the reversible agglutination of RBCs at cold temperature.^17,18^ However, hemolysis is extremely rare in MP pneumonia patients despite occasionally high CA titers, suggesting other trigger factors may be required for hemolysis in addition to RBC-binding autoantibodies and intact complements in patient sera.^19^ CAs-inducing immune reactions have also been observed in other conditions including Epstein–Barr virus or adenovirus infections, autoimmune diseases such as systemic lupus erythematosus, and malignancies such as lymphoma.^18,20–22^

Regarding infectious diseases, the host immune system may control not only the substances from the infectious pathogens, including pathogen-associated molecular patterns (PAMPs), but also those from the injured host cells caused by infectious insults, including damage (or danger)-associated molecular patterns (DAMPs).^23,24^ Furthermore, intact MPs are difficult to find in pulmonary lesions as well as in extrapulmonary lesions such as encephalopathy but are occasionally positive on polymerase chain reaction.^25^ Therefore, the substances that induce CAs may not originate from MPs or other pathogens but rather from the same kind of host cells affected by insults from MP infection or other conditions (i.e., a kinds of DAMPs?). Moreover, the substances inducing extrapulmonary manifestations in MP infection or other respiratory pathogen infections could be postulated to function in the same way.^23^

In the present study, MP IgM antibody titers and CAs titers were correlated during the acute stage of the disease; when CAs titers were matched to one-tenth of MP IgM titers. Other studies also report the association between MP-specific antibodies and CAs, suggesting CAs testing can be used as a screening test for MP infection. The sensitivities of a single CAs test in MP infection range 50% to 100%.^11–13^ As CA titers ≥1:32 or ≥1:64 are rarely observed in other viral infections, this cutoff has been proposed to be helpful for the diagnosis of MP pneumonia. It was previously recommended that repeated examinations could be performed every other day in the early stage of MP pneumonia.^18^ In the present study, 31.3% (131/418) and 62% (259/418) of the patients had titers ≥1:32 at presentation and the 2nd examination, respectively. A titer ≥1:32 at presentation and identification of change of CA titers during hospitalization showed that the corresponding rates of the paired CAs tests were 81% to 96% in subgroups of MP pneumonia patients diagnosed by MP-specific IgM antibodies. To our knowledge, this is the 1st report regarding availability of the paired CAs tests for MP infection.

It is well known that single serologic test has limited values for early diagnosis of MP pneumonia because of no IgM antibodies in the early stage of MP infection and long-term persistence of IgM antibodies after MP infection.^1–3^ In the present study, approximately one-third of patients were negative for MP-specific antibodies (33.4%, 148/418) and CAs (37.6%, 157/418) at presentation. Patients admitted earlier tended to exhibit a higher rate of negative titers and lower titers; among the patients with a 7-day fever duration, 22.9% (20/89) showed a negative titer (≤1:40) and small part of patients had longer duration for seroconversion. Similar findings were also observed with regard to CAs titers. A single high MP-specific antibody titer (e.g., ≥1:320) has been regarded as a diagnostic marker of MP pneumonia, but only 25.8% of patients (108/418) had such a titer at admission, whereas 72.2% of patients (304/418) had the titer (≥1:320) at the 2nd examination. On the other hand, paired MP specific-IgG serologic tests 2 to 4 weeks apart have also some problems because of difficulty of sample collection or no reliable test kits of higher sensitivity and specificity on MP-specific IgGs.^7,10,26,27^ Therefore at present time, short-term follow-up confirmation of seroconversion or increased titers of specific IgM antibody may be essential for the definitive serologic diagnosis of MP pneumonia.

It is believed that many kinds of MP-specific antibodies are produced during MP infection. Although various serologic methods have been developed, including complement fixation assay, indirect immunofluorescence assay, indirect microparticle agglutination assay (i.e., Serodia Myco II), enzyme-linked immunosorbent assay (ELISA), and enzyme-linked immunoassay, the antigenic compositions used in these serologic diagnostic kits differ among manufacturers. Therefore, the positivity rates of MP-specific IgM and IgG antibodies determined by each method can differ significantly during the acute stage of MP infection.^26,27^ In addition, MP-specific IgM antibodies can appear prior to CAs, and MP-specific IgM antibody titers can peak in the early stage of the disease as shown in the present study. Although appearance of CAs may be a nonspecific reaction against MP pathogen-components, CAs may reflect a systemic reaction against the insults from MP infection during MP pneumonia epidemics. Therefore, a short-term paired CAs test may be used as an adjuvant serologic test for the diagnosis of the MP infection, especially on evaluation for old adult patients who may be suspected to have a reinfection and show rare MP specific IgM response in the acute stage during MP pneumonia epidemics.^28,29^ This paired CAs method may also be used for a base on the sensitivity of newly developed diagnostic kits even at the present time.^30,31^

This study may have some limitations. Although we showed the changes of MP-specific and nonspecific IgM antibodies within a mean of 3.4 days in the early stage of the disease, this method also has a limitation of early diagnosis for the decision of suitable antibiotic. Additionally, the microparticle agglutination assay used in the present study is also reported to produce results slightly different from those of other serologic analyses,^26,27^ suggesting that more reliable serologic methods are needed in the future.

In conclusion, we showed that MP specific IgM and nonspecific IgM antibodies (CAs) were seroconverted or increased antibody titers within several days. Therefore, paired or repeated MP-specific IgM tests in the acute stage and early convalescent stage (during hospitalization) may be used as a confirmative diagnostic method for MP infection studies. In addition, CAs titers are correlated with the titers of MP-specific antibodies in MP pneumonia patients and show diagnostic value of exceeding 80% in paired examinations. Paired or repeated CAs tests in the acute stage may also be used as an adjuvant diagnostic method for MP infection, especially in MP pneumonia epidemics, given the current unavailability of a perfect serologic test.
